# Supplementary material for: Structural Characterization of, and Protective Effects Against, CoCl2-Induced Hypoxia Injury to a Novel Neutral Polysaccharide from Lycium barbarum L
Source: Foods. 2025 Jan 21;14(3):339. doi: 10.3390/foods14030339 (PMC11818000; doi:10.3390/foods14030339)
Supplement: Supplementary file 1 [file foods-14-00339-s001.zip › foods-3318343-supplementary.docx]

Structural characterization and protective effects against CoCl_2_-induced hypoxia injury of a novel neutral polysaccharide from *Lycium barbarum* L.

Yunchun Li ^1,†^, Jianfei Liu ^1,†^, Dong Pei ^1^, Duolong Di^1,^*

^1^ CAS Key Laboratory of Chemistry of Northwestern Plant Resources and Key Laboratory for Natural Medicine of Gansu Province, Lanzhou Institute of Chemical Physics, Chinese Academy of Sciences, Lanzhou 730000, China; jfliu@licp.cas.cn

***** Correspondence: didl@licp.cas.cn

**†** These authors contributed equally to this work.

**Table S1.** Primer list

| Primer | Sequence (5' to 3') | Length（bp） |
| --- | --- | --- |
| Actin-F  Actin-R | AGACCTTCAACACCCCAGCCA ATGGGCACAGTGTGGGTGACC | 121 |
| Bax-F  Bax-R | GTCTGCGGGGAGTCACG ACCCTGTAGCAAAAAGGCCC | 159 |
| Bcl2-F  Bcl2-R | GGTGAACTGGGGGAGGATTG ACTCGCTCAGCTTCTTGGTG | 102 |
| CAT-F  CAT-R | ACTCACCTGAAGGACCCTGA GCCATTCATGTGCCGATGTC | 123 |
| Casp3-F | GGAGCTTGGAACGCGAAGA | 169 |
| Casp3-R | ACACAAGCCCATTTCAGGGT |  |
| Gpx1-F | AGTGCGAGGTGAATGGTGAG | 125 |
| Gpx1-R | CACCGGGGACCAAATGATGT |  |
| HIF-1α-F | CATTGGCATGGAAGGAAATGTG | 133 |
| HIF-1α-R | GACAGGGTATGGATAGAGGCA |  |
| SOD1-F  SOD1-R | GCTTCTGTCGTCTCCTTGCT CTCGAAGTGAATGACGCCCT | 133 |
| VEGF-F | CGGGCCTCTGAAACCATGAA | 122 |
| VEGF-R | GCTTTCTGCTCCCCTTCTGT |  |

**Table S2.** Methylation analysis of LICP009-3F-1a

| Methylated sugar | Type of linkage | Molar ratio (%) |
| --- | --- | --- |
| 1,4-di-O-acetyl-2,3,5-tri-O-methyl arabinitol | T-Ara*f* | 8.9 |
| 1,5-di-O-acetyl-2,3,4,6-tetra-O-methyl glucitol | T-Glc*p* | 6.9 |
| 1,5-di-O-acetyl-2,3,4,6-tetra-O-methyl galactitol | T-Gal*p* | 10.2 |
| 1,2,5-tri-O-acetyl-3,4-di-O-methyl xylitol/1,4,5-tri-O-acetyl-2,3-di-O-methyl xylitol | 1,2/4-Xyl*p* | 5.5 |
| 1,3,5-tri-O-acetyl-2,4,6-tri-O-methyl galactitol | 1,3-Gal*p* | 5.8 |
| 1,5,6-tri-O-acetyl-2,3,4-tri-O-methyl mannitol | 1,6-Man*p* | 3.1 |
| 1,5,6-tri-O-acetyl-2,3,4-tri-O-methyl glucitol | 1,6-Glc*p* | 4.8 |
| 1,4,5-tri-O-acetyl-2,3,6-tri-O-methyl glucitol | 1,4-Glc*p* | 11.3 |
| 1,5,6-tri-O-acetyl-2,3,4-tri-O-methyl galactitol | 1,6-Gal*p* | 16.9 |
| 1,3,5,6-tetra-O-acetyl-2,4-di-O-methyl galactitol | 1,3,6-Gal*p* | 26.6 |


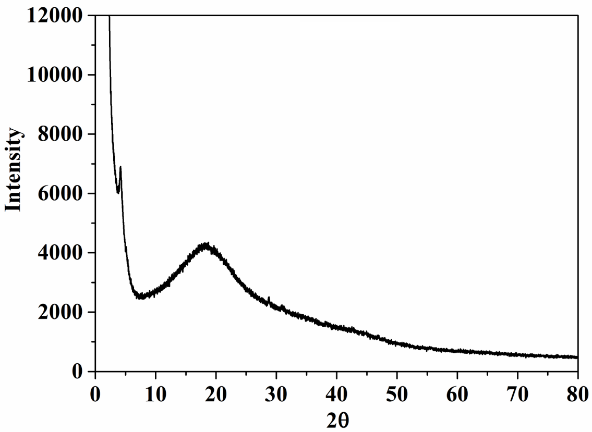


**Fig. S1** XRD pattern of LICP009-3F-1a


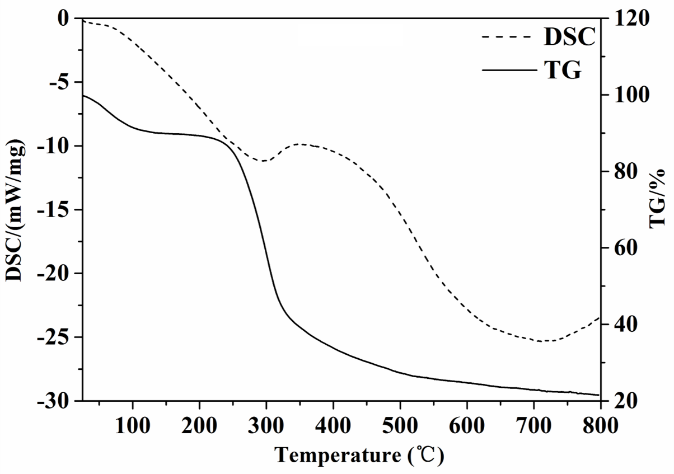


**Fig. S2** TGA and DCS trace of LICP009-3F-1a


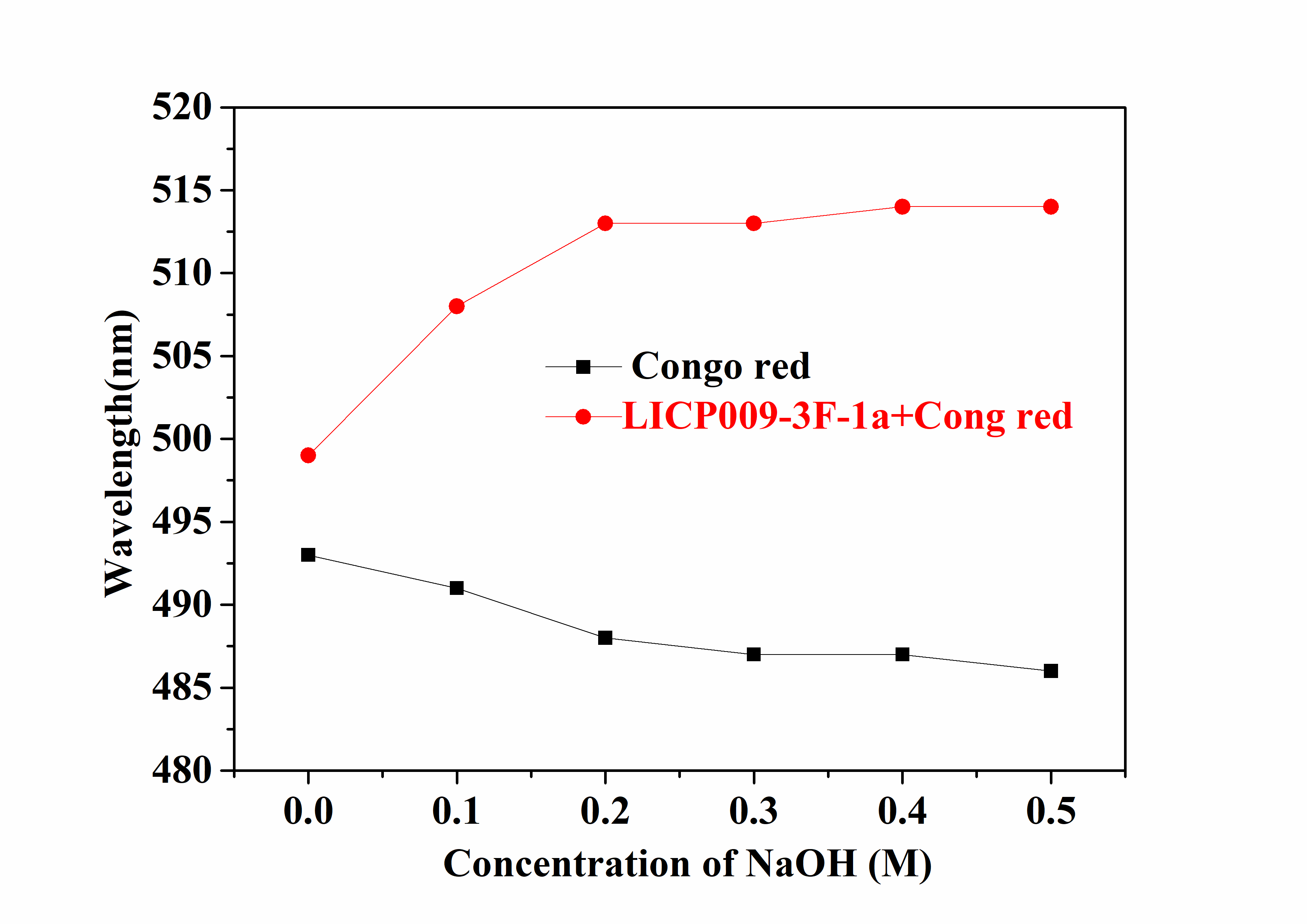


**Fig. S3** Maximum absorption wavelengths of Congo red and the Congo-red/LICP009-3F-1a complex as functions of NaOH concentrations


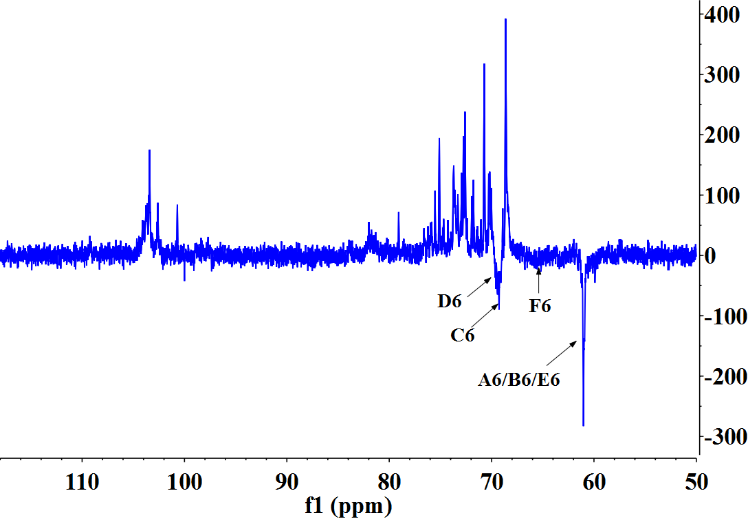


**Fig. S4** DEPT135 spectra of LICP009-3F-1a
